# Molecular Imaging of a New Multimodal Microbubble for Adhesion Molecule Targeting

**DOI:** 10.1007/s12195-018-00562-z

**Published:** 2018-11-28

**Authors:** Mona Ahmed, Björn Gustafsson, Silvia Aldi, Philip Dusart, Gabriella Egri, Lynn M. Butler, Dianna Bone, Lars Dähne, Ulf Hedin, Kenneth Caidahl

**Affiliations:** 1 Department of Molecular Medicine and Surgery, Center for Molecular Medicine, Karolinska Institutet, 17176 Stockholm, Sweden; 2 Section for Medical Inflammation Research, Department of Medical Biochemistry and Biophysics, Karolinska Institutet, 17177 Stockholm, Sweden; 3 Department of Cellular and Clinical Proteomics, Kungliga Tekniska Högskolan (KTH), Science for Life Laboratory, 17165 Stockholm, Sweden; 4 Surflay Nanotec GmbH, Max-Planck-Straße 3, 12489 Berlin, Germany; 5 Clinical Chemistry and Blood Coagulation Research, Department of Molecular Medicine and Surgery, Karolinska Institutet, 17176 Stockholm, Sweden; 6 Department of Molecular and Clinical Medicine, Institute of Medicine, Sahlgrenska Academy, University of Gothenburg, 41345 Gothenburg, Sweden

**Keywords:** Antibodies, Contrast agent, Confocal microscopy, Endothelial cells, Flow cytometry, Inflammation, *In vitro*, *In vivo*, Macrophages, Polyvinyl-alcohol

## Abstract

**Introduction:**

Inflammation is an important risk-associated component of many diseases and can be diagnosed by molecular imaging of specific molecules. The aim of this study was to evaluate the possibility of targeting adhesion molecules on inflammation-activated endothelial cells and macrophages using an innovative multimodal polyvinyl alcohol-based microbubble (MB) contrast agent developed for diagnostic use in ultrasound, magnetic resonance, and nuclear imaging.

**Methods:**

We assessed the binding efficiency of antibody-conjugated multimodal contrast to inflamed murine or human endothelial cells (ECs), and to peritoneal macrophages isolated from rats with peritonitis, utilizing the fluorescence characteristics of the MBs. Single-photon emission tomography (SPECT) was used to illustrate ^99m^Tc-labeled MB targeting and distribution in an experimental *in vivo* model of inflammation.

**Results:**

Flow cytometry and confocal microscopy showed that binding of antibody-targeted MBs to the adhesion molecules ICAM-1, VCAM-1, or E-selectin, expressed on cytokine-stimulated ECs, was up to sixfold higher for human and 12-fold higher for mouse ECs, compared with that of non-targeted MBs. Under flow conditions, both VCAM-1- and E-selectin-targeted MBs adhered more firmly to stimulated human ECs than to untreated cells, while VCAM-1-targeted MBs adhered best to stimulated murine ECs. SPECT imaging showed an approximate doubling of signal intensity from the abdomen of rats with peritonitis, compared with healthy controls, after injection of *anti*-ICAM-1-MBs.

**Conclusions:**

This novel multilayer contrast agent can specifically target adhesion molecules expressed as a result of inflammatory stimuli *in vitro*, and has potential for use in disease-specific multimodal diagnostics *in vivo* using antibodies against targets of interest.

**Electronic supplementary material:**

The online version of this article (doi:10.1007/s12195-018-00562-z) contains supplementary material, which is available to authorized users.

## Introduction

A number of imaging platforms are available for molecular imaging, including optical imaging, positron- or single-photon emission tomography (PET or SPECT), magnetic resonance imaging (MRI), and contrast-enhanced ultrasound (CEUS). SPECT and PET are usually combined with computed tomography (CT) as so-called hybrid imaging,^19^ and the combination of PET/MRI is also used clinically.^34^ Ultrasound (US) remains the most widespread technique for diagnostic imaging although research on its combination with other techniques is ongoing.^22^,^37^ Together, these imaging capabilities provide the basis for *in vivo*, multimodal, and targeted molecular imaging.^20^,^25^,^32^,^36^ Each imaging platform is dependent on specific imaging moieties, such as superparamagnetic iron oxide nanoparticles (SPIONs), ^99m^Tc, ^18^F, or microbubbles (MBs). Molecular imaging enables the study of biological processes such as inflammation, thrombosis, phagocytic activity, metabolic activity, lipid uptake, oxidative stress, proteinase activity, neoangiogenesis, osteogenesis, and thrombosis, each with specific molecular targets.^59^

Focusing on inflammation, there is an urgent need for new imaging probes for use with the numerous modalities now available for imaging of inflammation-driven diseases,^18^,^43^,^72^ including cancer and atherosclerosis. Vascular inflammation is an important trigger for recruiting inflammatory cells such as leukocytes, e.g., lymphocytes, monocytes, and neutrophils. Early signs of vascular inflammation are characterized by elevated expression of cellular adhesion molecules (CAMs) and selectins on the vascular endothelial cells lining the vessels.^42^ Because adhesion molecules such as CAMs and selectins interact to mediate, recruit, and facilitate cellular responses to inflammation, they are strong potential targets for new imaging probes or therapeutic agents.^59^ Efforts have been made to identify adhesion molecules using CEUS^36^,^39^ and other techniques. Macrophages have been identified as one of the most important early cellular components in the progression of inflammation,^42^ and thus, are also interesting as strategic targets. Although PET imaging using ^18^F-fluorodeoxyglucose (^18^F-FDG) is most widely used to evaluate the density of macrophages in atherosclerosis, it has a number of limitations.^31^ Established diagnostic tools for imaging of signs of early inflammation and cellular and molecular processes are lacking.

It has recently been shown in both static and dynamic experiments that lipid- and polymer-based MBs conjugated to antibodies against ICAM-1, VCAM-1, and selectins have potential as targeted US and/or MRI contrast agents for endothelial cells.^10^,^73^–^75^ We previously investigated similar multimodal contrast agents, ~ 3 *µ*m sized air-filled polyvinyl alcohol (PVA) MBs,^2^,^6^,^7^,^12^,^14^,^15^,^23^,^64^,^69^ and found uptake by macrophages.^2^ Further development of this probe has been directed at specific molecular targeting of inflammatory cell markers. The MB evaluated in this report has a layer-by-layer (LbL) structure^23^,^69^ with a shell composed of layers of SPIONs, metal chelating ligands, and a fluorophore-labeled polyelectrolyte, and an outermost layer made of streptavidin. This construction enables the MBs to act as contrast or signal-providing agents for multiple imaging modalities, including US, MRI, SPECT/PET, and fluorescence imaging. The streptavidin surface also enables conjugation of biotinylated ligands, e.g., peptides or antibodies, for specific targeting towards molecules and cells; such as the CAMs and selectins expressed on the inflamed endothelium^10^,^38^ or macrophages present in inflammatory areas.

The purpose of this study was to evaluate the targeting efficiency, contrast distribution and imaging properties of the new contrast agent and its potential for specific adherence to inflammatory markers expressed on cell surfaces in models of inflammation in three different species using human venous and mouse arterial endothelial cells and rat *ex vivo* and *in vivo* models applying SPECT in the latter.

## MATERIALS AND METHODS

### Endothelial Cells

Mouse aortic endothelial cells (MoAoECs) were cultured in Endothelial Cell medium (CellBiologics Inc., Chicago, IL, USA). Pooled human umbilical vein endothelial cells (HUVECs) were cultured in Endothelial cell Medium-2 (EGM-2 BulletKit) (Lonza, Basel, Switzerland). Cells were maintained at 37 °C in a 5% CO_2_, 95% air humidified atmosphere and sub-cultured at a dilution of 1:3. All experiments were performed between passage 3 and passage 8.

Stimulation and starvation of endothelial cells were carried out in starvation medium for 2 h; consisting of basal growth medium with supplements and 0.5% fetal bovine serum (FBS). Cells were stimulated in starvation medium with 30 ng mL^−1^ of mouse recombinant tumor necrosis factor α (TNF-α) together with 40 ng mL^−1^ of mouse recombinant interleukin 1β (IL-1β) (R&D Systems, Bio-techne, Minneapolis, MN, USA), or 10 ng mL^−1^ human TNF-α alone for 6 or 24 h. Non-stimulated cells in starvation medium were used as control. The same cytokine stimulation protocol was used to study protein expression analysis by Western blot, flow cytometry and confocal imaging.

### Inflammation Model and Peritoneal Macrophages

Male Sprague–Dawley rats were maintained on a standard laboratory diet with continuous access to water and food, and kept in a controlled environment with a 12 h light and dark cycle. All institutional and national guidelines for the care and use of laboratory animals were followed and approved by the Stockholm Animal Ethics Committee. Peritonitis was induced by Zymosan A (100–140 mg kg^−1^ body weight, Sigma, Darmstadt, Germany) on the left side of the peritoneal cavity as previously described.^13^,^49^ The inflammatory response starts immediately and reaches its peak 1–4 days after injection.^13^,^21^ Preparation of the animals took place approximately 4 days prior to the SPECT imaging.

For isolation of peritoneal macrophages, rats were anesthetized 24 h post injection of Zymosan and the abdominal skin was opened using aseptic conditions followed by injection of 10 mL sterile 37 °C phosphate buffered saline (PBS) containing 1% penicillin–streptomycin (PEST) into the peritoneal cavity. The abdomen was gently massaged, and a small incision in the abdominal wall was made to collect the fluid under aseptic conditions. Cells were collected and cultured according to protocol.^78^ Adherent cells were cultured for maximum 1 day in DMEM/F12 medium (Gibco, Thermo Fisher Scientific), supplemented with 10% FBS, 1% sodium pyruvate and 1% PEST, and further characterized with macrophage markers (CD11b, EMR1)^78^ and examined for *anti*-ICAM-1-MB uptake by flow cytometry.

### Microbubbles

**Figure 1:**
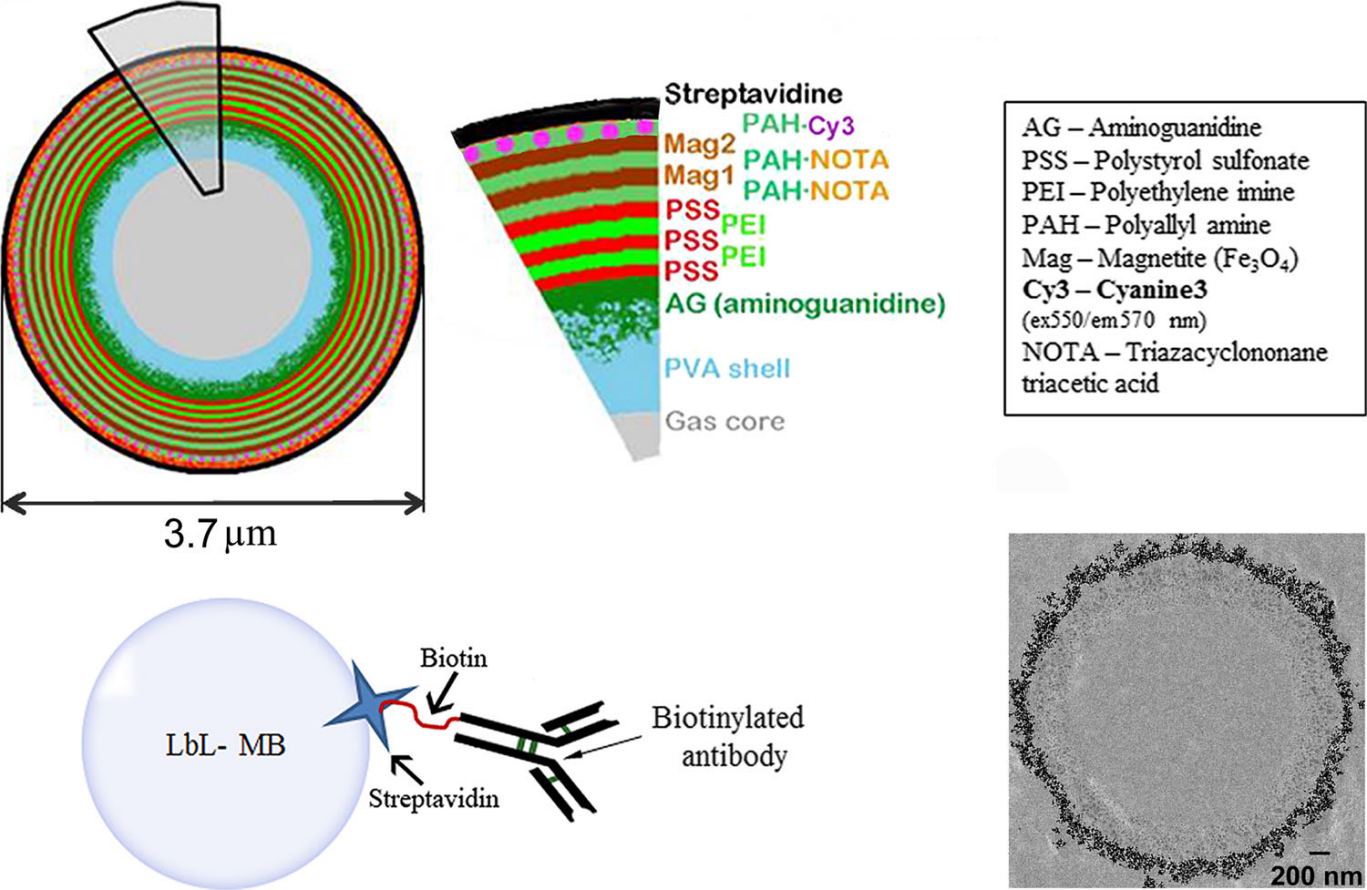
Schematic of the MB, layer by layer (upper panel), showing the different layers with potential for multimodal imaging. The layer characteristics of the MBs are listed (MB diameter 3.7 *µ*m in hydrated state as measured by AFM). MBs can be coupled to different antibodies *via* a streptavidin–biotin linkage (lower left panel). TEM (lower right panel) image of a MB with two layers of SPIONs as used in this study.

Basic PVA-MBs were made using a typical method from periodate activated PVA as described in the literature.^14^ Multimodal MBs used in all experiments were manufactured using LbL technology (Surflay GmbH, Berlin, Germany).^56^ This multimodal MB was developed as a part of a European Union project and further characterized and assessed for imaging and targeting properties. The PVA-MB surface was functionalized by the introduction of a positive charge by the reaction with aminoguanidine hydrochloride, which then could be coated with alternating layers of polycations (poly(styrene sulfonate)/poly(ethylene imine)_2_) labeled with either metal chelation ligands (2-S-(4-isothiocyanatobenzyl)-1,4,7-triazacyclononane-1,4,7-triacetic acid, *p*-SCN-Bz-NOTA, Macrocyclics, Plano, TX, USA) or fluorochromes (Cy3^®^) and then two additional layers of citrate stabilized SPIONs (Fig. 1). The final layer of the shell consists of streptavidin (6 mg/m^2^, yielding approximately 2.8 × 10^6^ streptavidin molecules per MB) attached to a biotinylated layer of poly(allyl amine).

### Physical and Imaging Properties

Physical and imaging properties have previously been evaluated by transmission electron microscopy (TEM), US, *in vivo* fluorescence imaging (IVIS) and MRI by Barrefelt *et al.*^7^,^8^ Thereby, TEM demonstrated a microbubble diameter of 3.1 *µ*m.^7^ The morphology of the SPION MBs was also measured in our study by transmission electron microscopy (TEM, JEM-2100F, JEOL, Tokyo, Japan) and samples were prepared as previously described.^7^ The low frequency mechanics of the MBs, in dry and hydrated state, were evaluated by atomic force microscopy (AFM) yielding the diameter, shell thickness and the stiffness of the MBs.^57^ The high frequency mechanics of the MBs were measured by the backscattering power, attenuation coefficient and phase velocity using US.^12^

### Radio-Labeling

Microbubbles were radio-labeled just before use and prior to antibody conjugation; technetium-99 m, ^99m^Tc (T_½_ = 6.01 h), was used as the gamma emitting radionuclide.^68^ Labeling procedure was as follows; ^99m^Tc in the form of sodium pertechnetate (VII) was reduced to ^99m^Tc (IV) for chelation by the NOTA-ligand on the MB.^68^ The reduction was made by the addition of 8% v/v of sodium dithionite (10 mg mL^−1^ in 0.05 M Na_2_CO_3_), at pH 11, at room temperature (RT) for 5–10 min. Then 10^8^ MBs in 100 *µ*L of 1 M sodium acetate buffer, pH 8.3, was added and the pH adjusted to 4.5–5. The labeling reaction took 30–60 min at RT. The MBs were then purified from unbound metal ions by magnetic separation and washing a minimum of three times with sterile 0.9% saline solution. All reagents used were free from trace metals.

### Antibody Conjugation

To target the microbubbles against inflammation, biotinylated antibodies (Ab) were conjugated to the surface of the MBs by utilizing the extremely strong non-covalent interaction between streptavidin and biotin.^9^,^11^,^28^,^39^ Here 6 *µ*g of biotinylated antibody was added to 10^8^ MBs (approximately 240 × 10^3^ antibodies per MB) in 0.9% saline or PBS. Each antibody has a hydrodynamic radius of approximately 5.5 nm^33^,^67^ occupying an area of ~ 95 nm^2^ on the MB. If we estimate 2.8 × 10^6^ streptavidin molecules on each MB, around 12 streptavidin molecules would be available for each of the 240 × 10^3^ antibodies per MB. After antibody-conjugation MBs were purified from free unattached antibodies by washing in PBS. Biotinylated antibodies used are described further in the flow cytometry section.

### Enzyme-Linked Immunosorbent Assay and Bicinchoninic Acid Protein Assay

Enzyme-linked immunosorbent assay (ELISA) and bicinchoninic acid (BCA) protein assay was done to evaluate the amount of unattached biotinylated antibodies in the wash solution after antibody-conjugation to MBs. Corning Costa assay flat bottom half area plates were coated with recombinant mouse VCAM-1/CD106 Fc Chimera Protein, CF (R&D systems, Bio-techne, Minneapolis, MN, USA), at an approximate surface density of 111 × 10^3^*µ*m^−2^ and incubated at 4 °C over night. Plates were washed and blocked with 1% bovine serum albumin (BSA) for 1 h at RT. After blocking, plates were washed and samples were added to the plate (liquid from wash of MB-Ab-conjugation; 0, 4, 6, 12, 20 *µ*g biotinylated antibody (anti-VCAM-1)/10^8^ MB). Samples were incubated on plate for 2 h at RT. After incubation, plates were washed, and incubated with HRP-conjugated streptavidin 1:40 (R&D systems, Bio-techne, Minneapolis, MN, USA) for 2 h at RT. After washing, substrate solution (BioLegend, San Diego, CA, USA) was added to each well and incubated in darkness for 20 min at RT. Reaction was stopped by adding stop solution to each well. Plates were analyzed and read at 450 and 570 nm. The different washes from MB-Ab-conjugation representing 0, 4, 6, 12, 20 *µ*g/10^8^ MBs of added antibody were analysed for protein concentration of biotinylated antibody with Micro BCA™ Protein Assay Kit (Pierce, Thermo Fisher Scientific, Waltham, MA, USA) according to manufacturer´s protocol.

### Immunoblotting

Total cell lysates were prepared from MoAoECs and HUVECs in radioimmunoprecipitation assay (RIPA) (Thermo Fisher Scientific, Waltham, MA, USA) buffer with cOmplete™ ULTRA Tablets, Mini, EASYpack Protease Inhibitor (Sigma-Aldrich). Protein concentration was determined with Micro BCA^TM^ Protein Assay Kit (Pierce, Thermo Fisher Scientific, Waltham, MA, USA). Total protein lysates (25–30 *µ*g/lane) were prepared in 4× Laemmli buffer supplemented with β-mercaptoethanol and preheated (at 90 °C) for separation in SDS-PAGE 4–20% (Bio-Rad, Hercules, CA, USA). Polyvinylidene difluoride (PVDF) membranes (GE Healthcare Amersham Hybond^TM^-P) were probed with rabbit *anti*-mouse/human-ICAM-1, rabbit *anti*-mouse/human-VCAM-1 and rabbit *anti*-mouse/human-E-selectin (Santa Cruz Biotechnology, Dallas, TX, USA) at dilution of 1:500 overnight. HRP-labeled goat *anti*-rabbit and goat *anti*-mouse (Invitrogen, Thermo Fisher Scientific, Waltham, MA, USA) were used at dilution of 1:5000 and incubated at RT for 45 min. Human β-actin HRP-conjugated antibody (Alpha Diagnostic International, San Antonio, TX, USA) and mouse *anti*-α-tubulin were used as loading controls at dilutions of 1:20,000 and 1:1000 respectively. Proteins of interest were detected by Amersham ECL Prime Western Blotting Detection Reagent (GE Healthcare Life Sciences, Little Chalfont, UK).

### Flow Cytometry

Cytokine stimulated endothelial cells and control cells were washed twice with PBS, followed by trypsin digestion and collection by centrifugation at 185×*g* for 5 min. Cells were counted in a hemocytometer with Trypan Blue 0.4% (Gibco, Thermo Fisher Scientific), and re-suspended in 5% BSA in PBS, and blocked for 30–40 min on ice. Cells were then washed once with PBS prior to live-dead staining (LIVE/DEAD™ Fixable Aqua Dead Cell Stain Kit, for 405 nm excitation, Invitrogen, Thermo Fisher Scientific, Waltham, MA, USA). Cells were washed again with PBS and incubated in 1% BSA in PBS solution for 30–40 min with either fluorescent antibodies on a rocking platform (15 rpm), or antibody-conjugated-MBs under continuous rotation (15 rpm) of the tube to allow MB-cell interaction. Approximately 4 × 10^5^ cells were incubated with 8 × 10^6^ antibody-conjugated MBs in tubes with continuous agitation, allowing cells and MBs to interact. Cells were then washed three times with PBS and fixed in 4% paraformaldehyde (PFA). Cells were analyzed by FACS as percentage of Cy3 positive cells measured as median fluorescence intensities (MFI). All analyses were carried out within 48 h after fixation of cells. Experiments were repeated minimum 3–6 times with technical duplicates for analysis. Monoclonal fluorescent antibodies were used at a dilution of 1:50; PE hamster *anti*-mouse ICAM-1, PE rat *anti*-mouse VCAM-1, PE rat *anti*-mouse E-selectin, PE hamster IgG1κ isotype control, PE rat IgG2aκ isotype control, FITC mouse *anti*-rat ICAM-1, FITC mouse *anti*-human VCAM-1 (BD Biosciences, Franklin Lakes, NJ, USA), FITC mouse *anti*-human ICAM-1, FITC mouse *anti*-human E-selectin, FITC mouse IgG1κ isotype control (eBioscience, Thermo Fisher Scientific, Waltham, MA, USA). For the MB experiments the following biotinylated antibodies were used; hamster *anti*-mouse ICAM-1, rat *anti*-mouse VCAM-1, rat *anti*-mouse E-selectin, hamster IgG1κ isotype control, rat IgG2aκ isotype control, mouse *anti*-rat ICAM-1, mouse *anti*-human ICAM-1, mouse *anti*-human E-selectin, mouse IgG1κ isotype control (BD Biosciences, Franklin Lakes, NJ, USA) and mouse *anti*-human VCAM-1 (eBioscience, Thermo Fisher Scientific, Waltham, MA, USA), according to Microbubbles section. Alexa Fluor^®^ 700 mouse *anti*-rat CD11b (Bio-Rad, BioSite), PE-Cy5^®^ (PE/Cy5^®^ conjugation kit, Abcam, Cambridge, UK) labeled (according to the Conjugation Kit Protocol) rabbit *anti*-rat EMR1 (polyclonal) (Bioss Antibodies, Woburn, MA, USA), purified mouse *anti*-rat CD32 (rat BD Fc Block™) (1:100) (BD Biosciences, Franklin Lakes, NJ, USA). Flow cytometry analyses were performed on Dako Cytomation CyAn™ ADP Analyzer (Dako, Glostrup, Denmark), BD Influx™ and a BD LSRFortessa™ (BD Biosciences, Franklin Lakes, NJ, USA).

### Confocal Microscopy

Cells were plated in ibidi µ-slide VI^0.4^ chambers (ibidi GmbH, Martinsried, Germany) and grown to approximately 70–80% confluency, then stimulated with TNF-α and IL-1β for 24 h. After stimulation, cells were washed twice with PBS, blocked for 30–40 min in PBS with 5% goat serum, and then incubated with 2 × 10^6^ antibody-conjugated-MBs per 1 × 10^5^ cells for 1 h with the ibidi slides turned up-side down, allowing MB-cell interaction. Non-bound MBs were washed away and the cell nuclei were stained with 4′,6-diamidine-2′-phenylindole dihydrochloride (DAPI, Merck, Darmstadt, Germany), then washed and fixed in 4% PFA. A total of six Z-stack images, with a slice thickness of 1 µm, were taken per ibidi channel with a Leica TCS SP5 system (Leica Microsystems, Wetzlar, Germany) confocal microscope using a 60× oil immersion objective. Experimental set-up was repeated minimum 3 times for analysis.

### Flow Chamber Experiments

HUVECs were seeded on the upper surface of ibidi µ-slide VI^0.4^ chamber slides and cultured until confluence. Cells were stimulated with 10 ng mL^−1^ TNF-α and evaluated for *anti*-VCAM-1-MB attachment or *anti*-E-selectin-MB attachment after 6 h stimulation. MBs conjugated to mouse *anti*-IgG1κ (isotype control) were used as control. One end of the slide was connected to a 20 mL syringe placed in a syringe pump (Harvard Apparatus, Holliston, MA, USA). The other end was connected to a forked tube with one end in 37 °C medium, and the other to the microbubble solution. Clamps were used to block tubes at relevant time-points. The syringe pump withdrew at a fixed flow rate of 0.87 mL min^−1^, corresponding to a flow velocity of approximately 9.5 mm s^−1^ and a shear stress of approximately 2 dyn cm^−2^ at the cell surface. The cells were washed for 30 s in medium under flow, then microbubbles at a concentration of 2 × 10^6^ mL^−1^ were passed through the channels for 2 min. Videos of MB-attachment were recorded at 3 different fields of view per chamber. After 2 min the input was switched back to medium, and cells were washed with medium for 2 min at constant flow. An 8.7 mL min^−1^ pulse corresponding to approximately 20 dyn cm^−2^ was then applied for 5 s, to test the avidity of antibody-conjugated-MB to the cells. Still images were acquired at 3 fields of view per condition and at 4 time-points; both before and after MB flow, after wash, and after the 20 dyn cm^−2^-pulse. All image acquisition used a Nikon Ti microscope with heated chamber at 37 °C, and image acquisition handled by NIS elements software. Cells were then fixed with 4% PFA for 10 min at RT. Experimental set-up was repeated a minimum 4 times, and images were analyzed in Fiji ImageJ software.^63^ The number of MBs attached to cells, the number of cells, and the area in mm^2^ covered by cells were counted and analyzed manually using a cell counter plugin.^63^ For preparation of immunofluorescence imaging, fixed cells were blocked overnight with 5% goat serum at 4 °C. Cells were permeabilized and stained for actin with Alexa Fluor^®^ 488-conjugated phalloidin (Molecular Probes, Thermo Fisher Scientific, Waltham, MA, USA), and chambers were filled with 40 *µ*L DAPI-containing mounting medium (VECTASHIELD, Vector Laboratories, Burlingame, CA, USA). Confocal fluorescence images were taken as Z-stacks with a slice thickness of 1.2 *µ*m using a 20× water immersion objective on a Leica TPS SP5 confocal microscope, and were processed in Fiji image processing software,^63^ using a maximum intensity Z-projection.

### In Vivo SPECT Imaging

To further test targeting *in vivo* in inflamed tissue, an *in vivo* pilot study was performed. All imaging of the ^99m^Tc-labeled microbubbles was performed using a Siemens Symbia™16T human sized clinical SPECT/CT camera. 128 projection images were acquired on a 128 × 128 pixel matrix, pixel size 2.4 mm, either as a time series of planar images (dynamic) or tomographically (SPECT). In order to identify anatomical features, CT was performed in conjunction with SPECT. Transaxial slices were reconstructed from CT data (slice thickness 0.7 mm). SPECT data were reconstructed using a 3D method (Siemens Flash 3D) and the volume was then matched to the reconstructed CT volume. The radio-labeled microbubbles were typically injected intravenously (IV) as a slow infusion. A volume of 5 mL of MBs of 10^7^ mL^−1^ was infused over 1 h. The typical radioactive dose was approximately 15 MBq. Four rats with peritonitis were injected with *anti*-ICAM-1 conjugated MBs. The infusion was typically given 4–5 h before SPECT-imaging was done; one animal was imaged during the infusion. Additionally, three healthy rats were imaged as negative controls using the slow infusion protocol. Data analyses were performed by an independent investigator and done by drawing volumes of interests (VOI), using the CT-images, around the whole body and each organ (whole abdomen, left abdomen, right abdomen, liver, lungs, and kidneys), respectively. The signal in each VOI was then calculated as counts per ml tissue, and the differences in injected dose were compensated for by normalization to the counts per volume of the whole body. Results are reported as mean ± SD of normalized relative activity (NRA) per organ.

### Statistical Analysis

For the *in vitro* and *in vivo* studies we conducted a Shapiro–Wilk normality distribution test and proceeded with nonparametric Mann–Whitney *U* test for comparison of medians in GraphPad Prism for the *in vitro* studies. For the *in vivo* studies we conducted an unpaired *t* test with Welch’s correction for differences between the two animal groups (inflamed and healthy) and a paired t-test with Welch`s correction for differences within each individual. Statistical significance comparing medians or means was defined with a two-tailed *p* of < 0.05.

## RESULTS

### Characterization of Targeted Microbubbles

The morphological MB appearance is illustrated by a TEM image (Fig. 1). AFM measurements of MBs in dry state yielded a diameter of 3.1 ± 0.5 *µ*m and shell thickness of 253 ± 45 nm. In hydrated state the diameter was 3.7 ± 0.5 *µ*m with a shell thickness of 405 ± 69 nm and an average stiffness of 0.33 ± 0.13 N/m. Antibody-conjugation to MBs was optimized and microbubbles were purified from free antibody that had not attached to the MBs. Unattached antibodies were quantified by BCA protein assay and ELISA (Supplementary Fig. 1) showing that at higher amounts, ≥ 12 *µ*g/10^8^ MBs, the antibody binding is shown to be saturated. The saturation is reached somewhere between 6 and 12 *µ*g of added antibodies per 10^8^ MBs. In the following MB-experiments we used 6 µg biotinylated antibody for conjugation to 10^8^ MBs. Each MB (ø 3.7 µm based on AFM measurements) has an approximate surface area of 43.0 *µ*m^2^, yielding a maximum amount of antibodies fitting on the MB surface of 453 × 10^3^. Considering the results of the BCA assay and that we added 6 *µ*g (approximately 240 × 10^3^ per MB) of antibodies we could assume that 47% of the MB surface area was covered by antibodies, corresponding to 4900 antibodies/*µ*m^2^ or one antibody per 200 nm^2^. US-characteristics have also been evaluated, where the maximum backscattering power was determined to 16 dB at a concentration of 4.0 × 10^6^ MB/mL, the attenuation coefficient and phase velocity was determined to 0.35 dB/m and 1492 m/s respectively at the same MB concentration.

### Measurement of Endothelial Cell Activation

**Figure 2:**
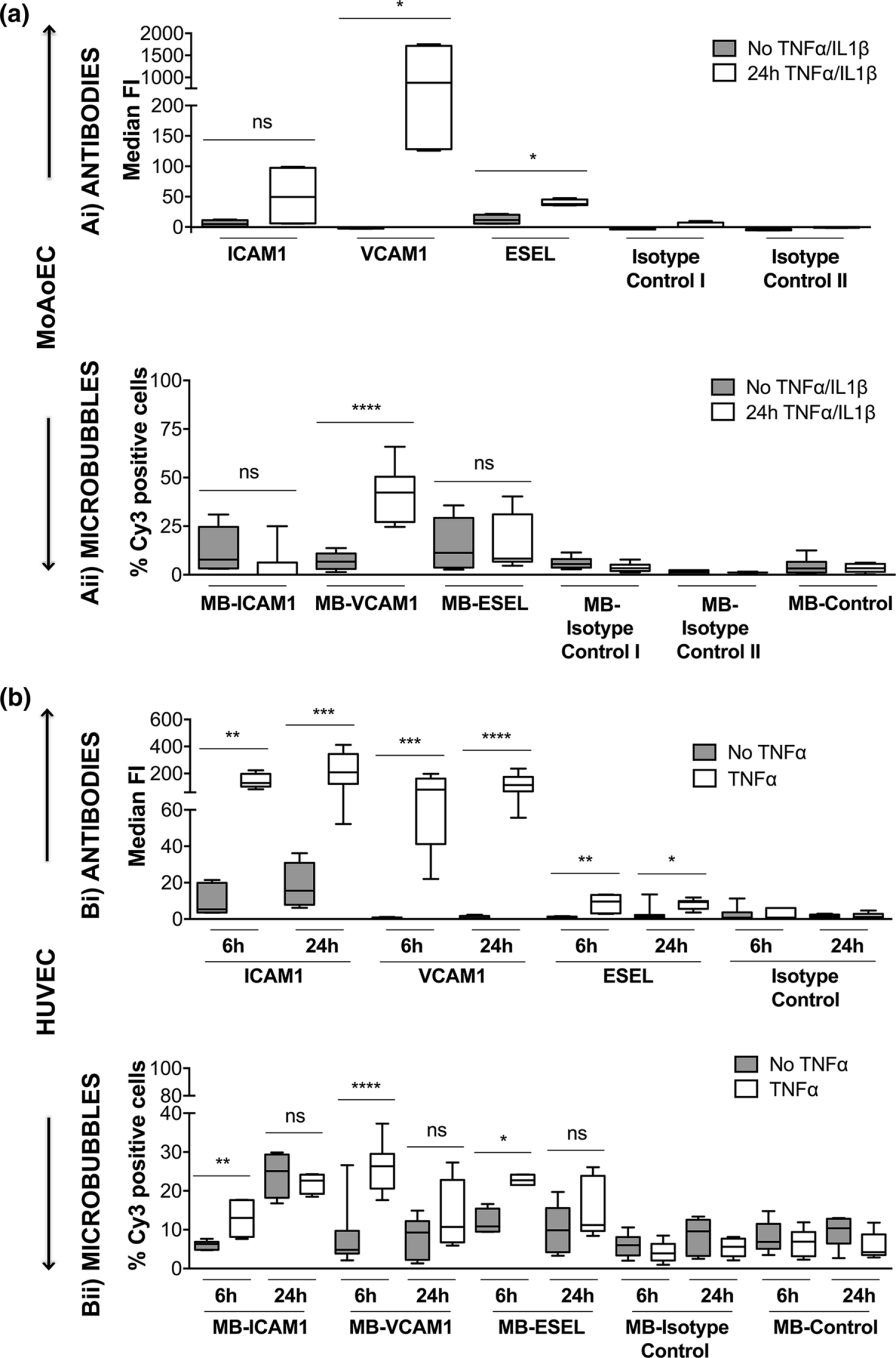
Targeting of surface adhesion molecules. Pooled flow cytometry data for (**a**) MoAoECs and (**b**) HUVECs, comparing non-treated and cytokine-treated cells. Antibody binding (i) was measured as median fluorescent intensity (MFI), and binding of antibody-labeled MBs (Cy3) (ii) was measured as % positive cells. Graphs are presented as box plots with median and interquartile range; whiskers represent minimum and maximum. Statistical significance was analyzed with Mann–Whitney *U* *p < 0.05 **p < 0.01 ***p < 0.001, ****p < 0.0001, *ns* non-significant. All targeted antibodies except *anti*-mouse ICAM-1 had significantly higher MFI than the relevant isotype controls (**ai**) PE–hamster IgG1κ (ns), PE–rat IgG2aκ (*p* < 0.05); (**bi**) FITC–mouse IgG1κ (*p* < 0.05–0.0001); and MB controls; MB-streptavidin (*p* < 0.05–0.0001) and MB-isotype controls (**aii**) biotin hamster IgG1κ (*p* < 0.05) and biotin rat IgG2aκ (*p* < 0.01–0.0001); (**bii**) biotin mouse IgG1κ (*p* < 0.05–0.0001) for all time points. Abbreviations: E-selectin (ESEL), isotype control I (hamster IgG1κ), isotype control II (rat IgG2aκ).

Western blots showed increased protein expression of the adhesion molecules (ICAM-1, VCAM-1, and E-selectin) upon cytokine stimulation of HUVECs and MoAoECs (Supplementary Figs. 2a and 2b). Surface expression of adhesion molecules upon cytokine stimulation was further confirmed *via* quantitative antibody-mediated flow cytometry (Fig. 2 and Supplementary Fig. 3). Flow cytometry data showed that antibody detection of ICAM-1, VCAM-1, and E-selectin on the surface of HUVECs was significantly upregulated after both 6 and 24 h of TNF-α stimulation compared with that in untreated cells and non-targeted controls (Fig. 2bi). In MoAoECs, only VCAM-1 and E-selectin showed significant differences in antibody binding and MFI between cytokine-stimulated cells compared to unstimulated and isotype controls (*p *< 0.05) (Fig. 2ai).

### Evaluation of Targeted MB Adhesion to Endothelial Cells

**Figure 3:**
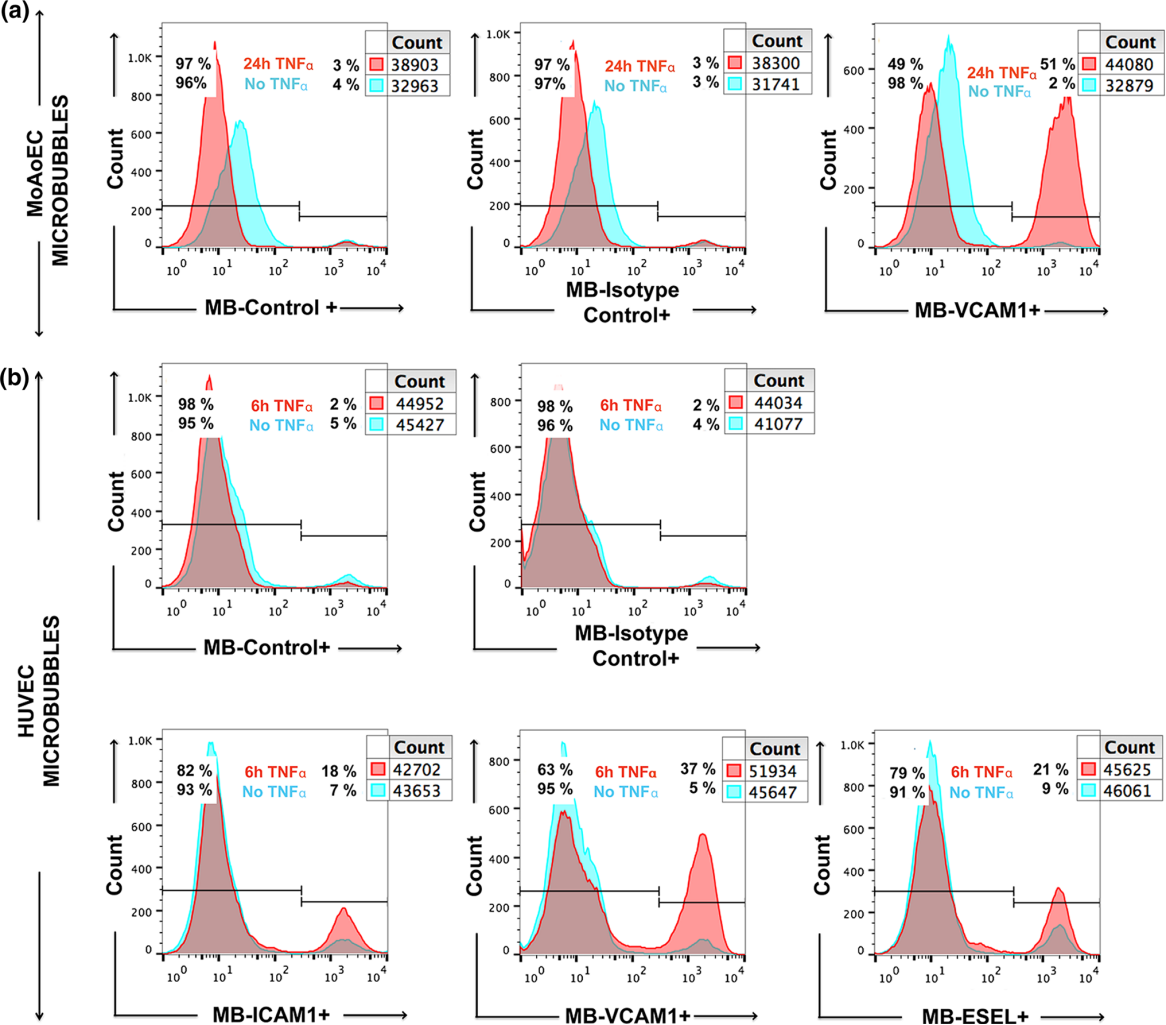
Flow cytometry histograms showing increased binding of antibody-labeled MBs to cytokine-treated (**a**) MoAoECs and (**b**) HUVECs measured as % Cy3 positive cells. *ESEL* E-selectin.

**Figure 4:**
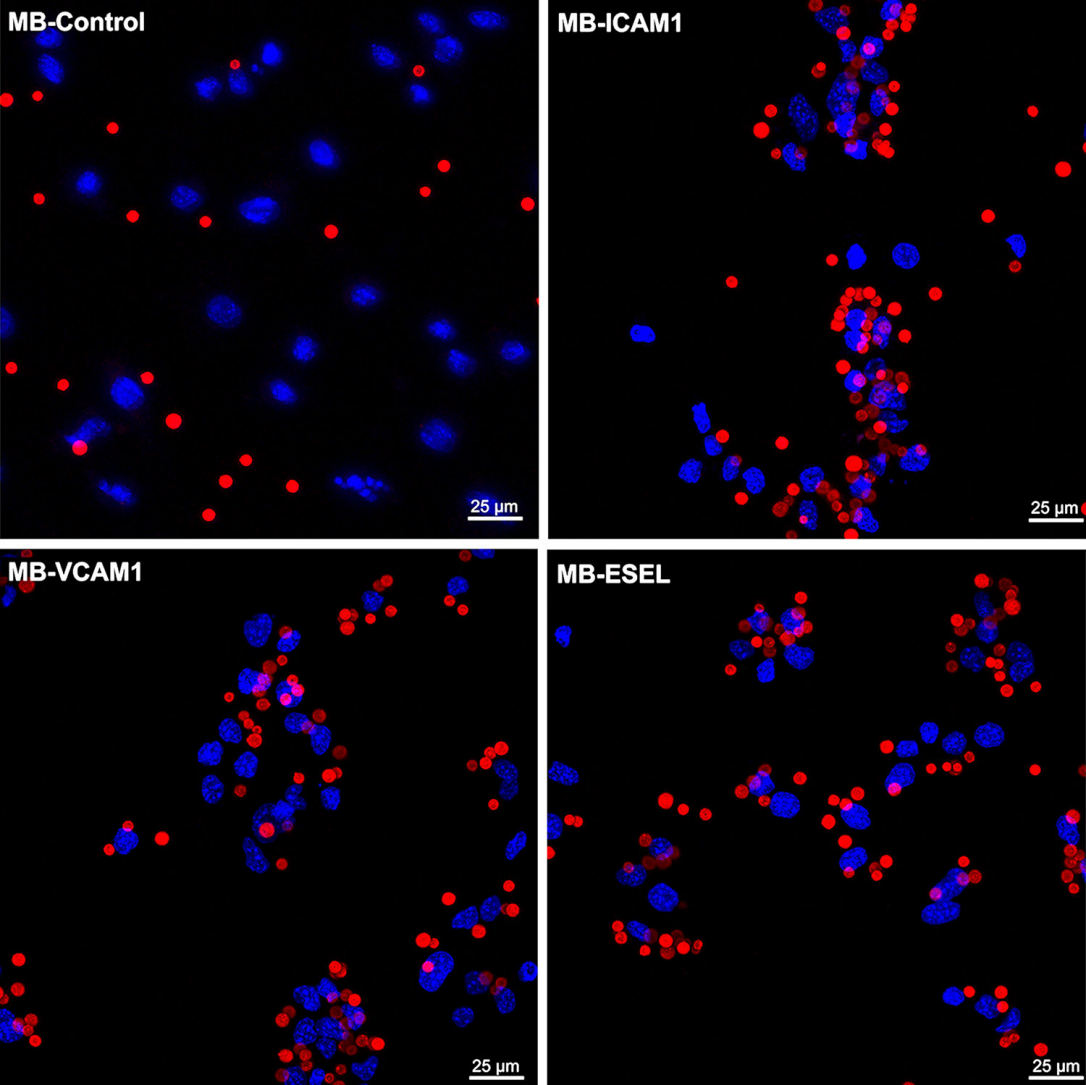
Maximum-intensity projection confocal laser scanning microscopy images of antibody-labeled MBs adhered to MoAoECs. Images show MBs (Cy3 red); MB-control (MB-streptavidin), *anti*-ICAM-1-MB, *anti*-VCAM-1-MB, and *anti*-E-selectin-MB adhered to endothelial cells (DAPI: blue nuclear staining). Images were acquired by Z-stack (step size 0.99 µm) with a 63× oil immersion objective. *ESEL* E-selectin.

We next investigated whether the binding of antibody-labeled MBs to activated cells followed the same pattern as that of antibodies alone. Quantitative flow cytometry showed up to sixfold (HUVEC) and 12-fold (MoAoEC) increases in targeted MBs bound to activated cells compared with control MBs (MB-streptavidin) or isotype controls at all time points (*p* < 0.05–0.0001) (Figs. 2aii and 2bii). *Anti*-VCAM-1-labeled MBs showed the greatest increase in adherence to activated cells in both MoAoECs and HUVECs *in vitro* (Figs. 2 and 3). Activated HUVECs showed a higher level of capture of ICAM-1-, VCAM-1-, and E-selectin-targeted MBs after 6 h of pro-inflammatory stimulation compared with untreated cells (*p* < 0.05–0.0001), while no significant difference was observed after 24 h of cytokine stimulation (Fig. 2bii). In MoAoECs, VCAM-1-targeted MBs showed a significant sixfold increase (*p* < 0.0001) in binding to 24-h cytokine-activated cells compared with untreated cells, although binding of ICAM-1- and E-selectin-targeted MBs was not increased (Fig. 2aii). Confocal images of MBs bound to the activated endothelial cells showed numerous targeted MBs (*anti*-ICAM-1, *anti*-VCAM-1, *anti*-E-selectin) clustering on 24-h cytokine-stimulated MoAoECs, with far fewer bound control MBs (Fig. 4). These results suggest that these antibody-labeled MBs have specific targeting properties for inflamed endothelial cells.

### Evaluation of MB Adhesion to Endothelial Cells Under Flow Conditions

**Figure 5:**
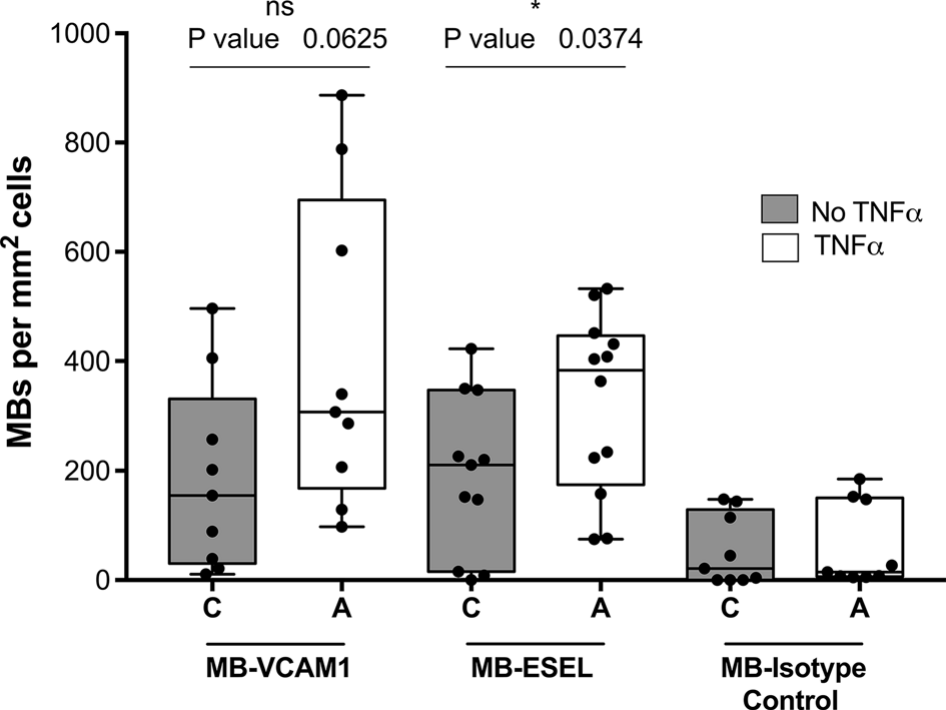
Pooled flow chamber experimental data for antibody-labeled MBs (VCAM-1, E-selectin; ESEL) binding to non-treated and TNF-α-treated HUVECs. Graphs show number of MBs bound per mm^2^ HUVECs surface area after 2 min continuous flow at 2 dyn cm^−2^ followed by washing at 2 dyn cm^−2^, and a 5-s pulse at 20 dyn cm^−2^. Graph presented as a box plot with median and interquartile range; whiskers represent minimum and maximum. Statistical significance was analyzed with Mann–Whitney *U* *p < 0.05, *ns* non-significant. Binding of all targeted MBs was significantly higher than that of MB-isotype control (mouse IgG1κ).

**Figure 6:**
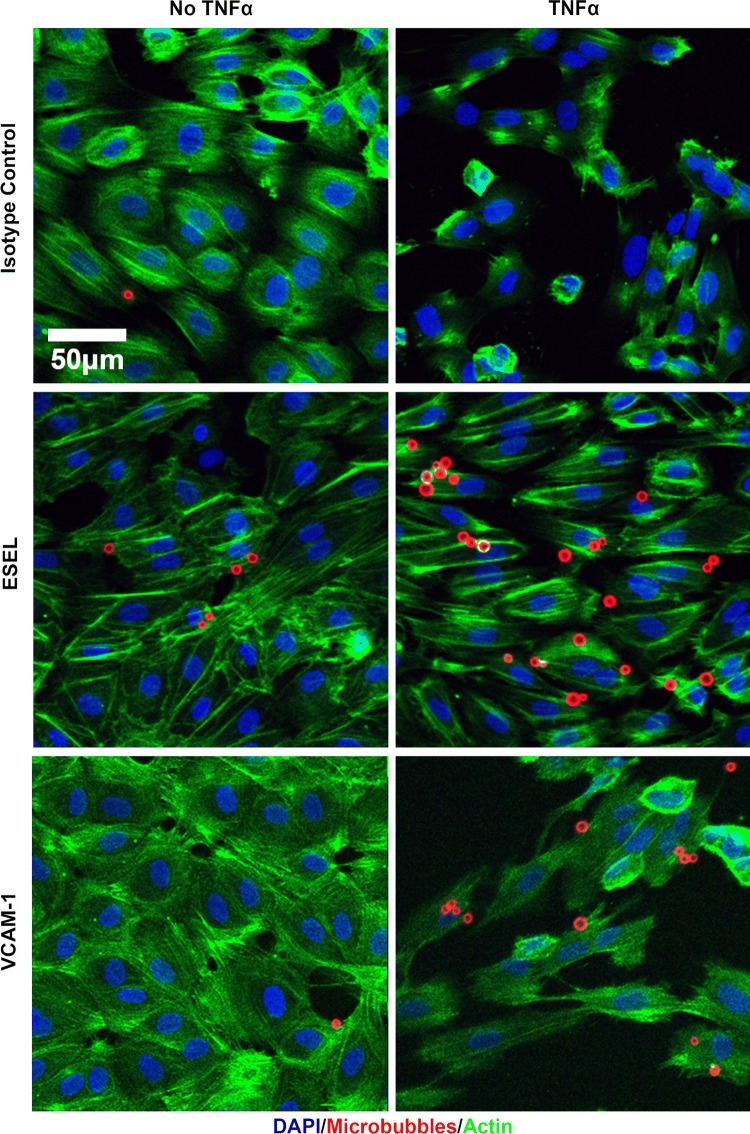
Maximum intensity projections of confocal laser scanning microscopy Z-stack images showing VCAM-1- and E-selectin (ESEL)-targeted MBs adhered to TNF-α-treated HUVECs compared with untreated cells and non-targeted isotype control-MBs (mouse IgG1κ). Images were acquired after 2 min continuous flow at 2 dyn cm^−2^ followed by wash and a 20 dyn cm^−2^ pulse. Blue staining represents DAPI staining of cell nuclei; red represents Cy3 antibody-labeled MBs; green represents Alexa Fluor 488-phalloidin staining of actin.

For a more clinically relevant MB setup and to mimic better a physiological MB–EC interaction, we measured MB binding to HUVEC-coated chamber slides under flow conditions. Based on their superior binding in the flow cytometry data, we chose to test the flow conditions using only the *anti*-E-selectin and *anti*-VCAM-1 antibody-labeled MBs. HUVECs were stimulated with TNF-α for approximately 6 h to obtain maximum expression levels of VCAM-1 and E-selectin, and the cells were then exposed to antibody-coated MBs under 2 dyn cm^−2^ shear stress (Supplementary video). Quantitative data showed firm adhesion of targeted MBs to HUVECs even after applying a high 20 dyn cm^−2^ shear stress pulse corresponding to the mean arterial hemodynamic shear stress,^45^,^53^,^55^ suggesting stronger adherence of targeted MBs to the cells compared with non-targeted isotype-control MBs (IgG1κ) (Fig. 5). MBs coated with *anti*-E-selectin showed significantly increased binding to activated HUVECs compared with untreated cells (*p* = 0.0374), and *anti*-VCAM-1-coated MBs tended to show increased binding (*p* = 0.0625) (Fig. 5). Isotype (IgG1κ)-coupled MBs had significantly weaker binding to activated HUVECs compared with targeted MBs (*p* = 0.0012 compared with VCAM-1 and *p* = 0.0003 compared with E-selectin). Confocal images of MB binding to HUVECs after flow illustrated the attachment of antibody-coated MBs to endothelial cells (Fig. 6). The number of bound MBs was consistent with the results of static experiments.

### Ex Vivo MB Uptake in Peritoneal Macrophages

**Figure 7:**
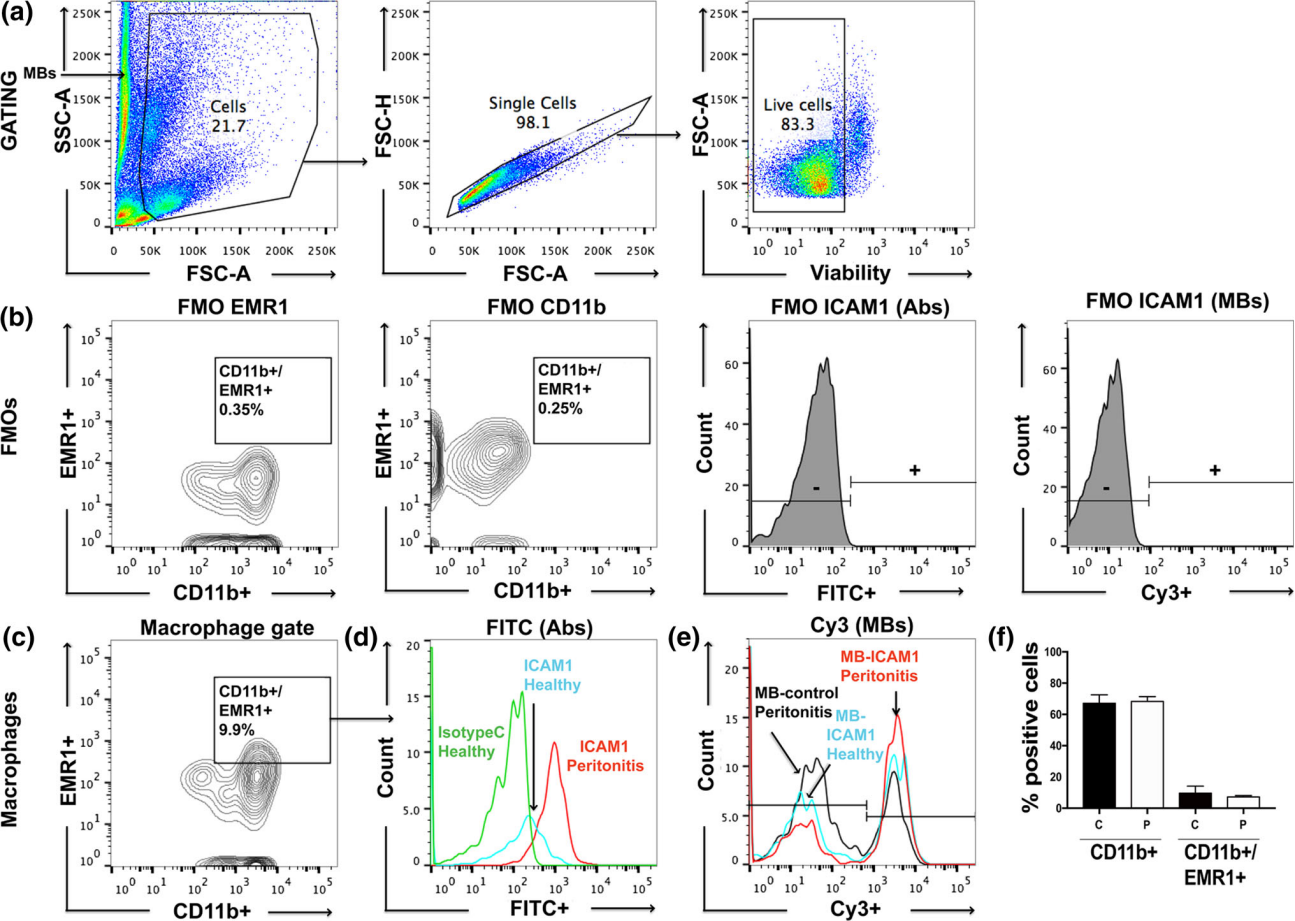
Flow cytometry data showing ICAM-1-MB targeting to peritoneal macrophages isolated from healthy control rats and rats with Zymosan-induced peritonitis. (**a**) Gating strategy for isolated peritoneal macrophages, (**b**) fluorescence minus one (FMO) controls, and (**c**) gating on CD11b + EMR1 + macrophages. Results are shown as histograms of (**d**) ICAM-1 targeted with fluorescent antibodies, or (**e**) antibody-labeled ICAM-1-MBs compared with control cells and non-targeted MBs. Green histogram: isotype control (FITC-mouse IgG1κ); black histogram: MB-control (Cy3 MB-streptavidin). Isolated macrophages from healthy rats (blue and green histograms) and peritonitis rats (red and black histograms). Cell counts in D and E were equal for each graph. Bar graph (**f**) shows the cell population of CD11b + and double positive cells (CD11b + EMR1 +) in healthy (**c**) and peritonitis (P) rats. Sample size n = 3 per group.

To examine whether the antibody-labeled MBs could target macrophages, we isolated peritoneal macrophages from a rat model of peritonitis and healthy control rats to compare the uptake of *anti*-ICAM-1-MBs in inflamed and non-inflamed cells. Cell characterization showed subpopulations of CD11b + and double positive cells (CD11b + EMR1 +) in our flow cytometry data set (Fig. 7f). These cells were then gated for ICAM-1-positive cells (Figs. 7a–7c). Results show a higher capture of *anti*-ICAM-1-antibody and *anti*-ICAM-1-MBs by peritoneal macrophages (CD11b + EMR1 + cells) from rats with inflammation compared with macrophages from healthy rats and non-targeted control MBs (FITC isotype control and MB-streptavidin) (Figs. 7d and 7e). Macrophages isolated from rats with peritonitis showed a 23% increase in the MFI of the Cy3 signal for *anti*-ICAM-1-MB compared with macrophages from healthy rats, and a 46% increase compared with control MBs (MB-streptavidin) (Fig. 7e).

### In Vivo Imaging of Radio-Labeled MBs in Experimental Inflammation

**Figure 8:**
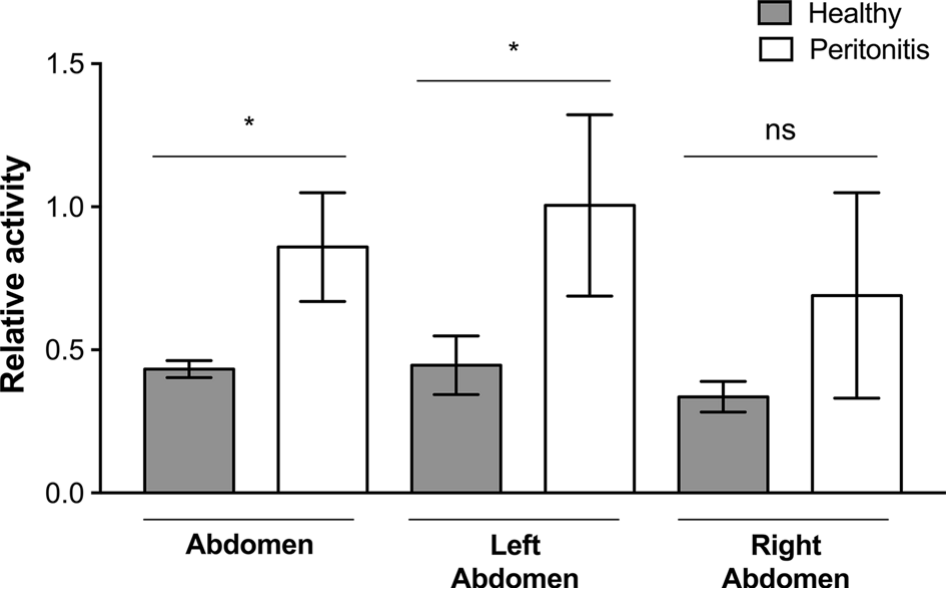
SPECT-signals from inflamed and healthy control rats infused with ^99m^Tc-labeled *anti*-ICAM-1-MB: Bar plot of the mean normalized relative activities (NRA) in the abdominal area in control (n = 3) vs. inflamed peritonitis (n = 4) rats; whiskers represent standard deviation. Comparison of healthy and inflamed animals: abdomen *p *= 0.0188, left abdomen *p *= 0.0323; right abdomen *p *= 0.1418. Significance of differences between the inflamed and control groups was analyzed using an unpaired *t*-test with Welch’s correction *p < 0.05. No significant differences were observed between left and right sides of the abdomen within each individual: inflamed animals *p *= 0.3121, healthy animals *p *= 0.1902. Statistical analysis with paired *t*-test with Welch’s correction *p < 0.05.

**Figure 9:**
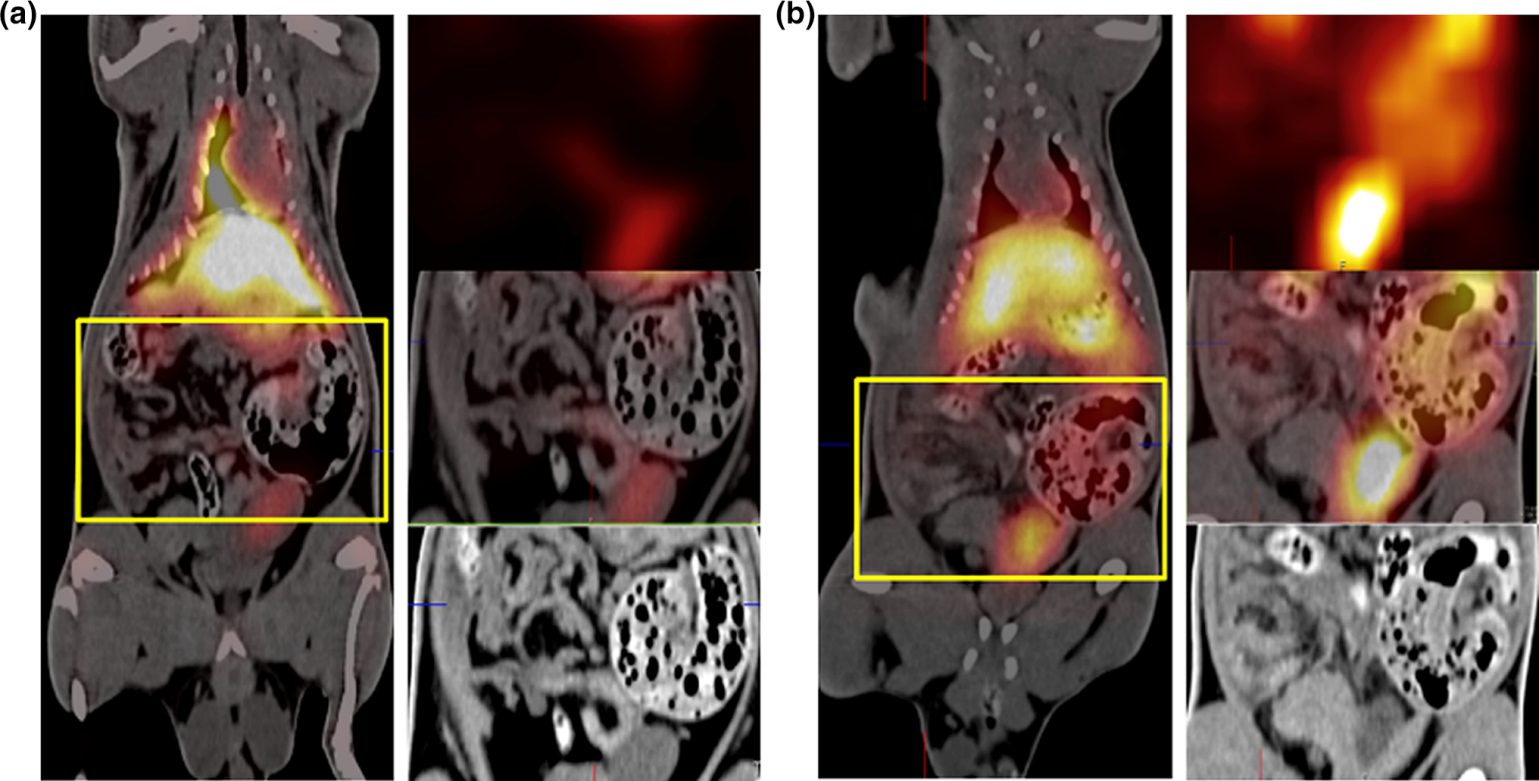
Whole-body SPECT/CT images of rats infused with ^99m^Tc-labeled *anti*-ICAM-1-MB contrast agent. The yellow rectangle indicates the abdominal area. To the right are three enlargements of this area showing the SPECT image (top), the CT image (bottom), and the SPECT/CT overlay (middle). (**a**) Healthy rat without inflammation, and (**b**) rat with bowel inflammation (peritonitis).

**Figure 10:**
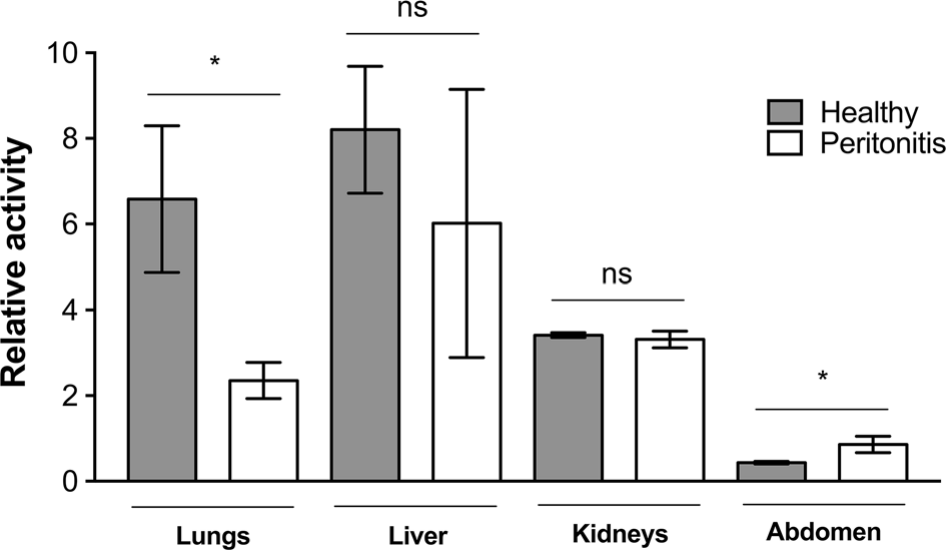
Organ distribution of ^99m^Tc-labeled *anti*-ICAM-1-MBs *in vivo* as quantified from SPECT/CT-images. Plotted are the mean normalized relative activities (NRA) for each organ. Significance of differences between the inflamed (n = 4) and control (n = 3) groups were analyzed with an unpaired *t*-test with Welch’s correction *p < 0.05; lungs *p *= 0.0452, liver *p *= 0.2815, kidneys *p *= 0.4012 and abdomen *p *= 0.0188; whiskers represent standard deviation.

To examine the ability of the targeted MBs to attach to inflamed areas *in vivo*, a pilot imaging study was performed. Four rats with peritonitis and three healthy control rats were infused with *anti*-ICAM-1 antibody-coated MBs radiolabeled with gamma-emitting ^99m^Tc for SPECT imaging. The injection strategy used was a slow infusion over 1 h to minimize lung entrapment.^41^ Results from the SPECT/CT imaging experiments showed a mean NRA of 0.86 in the abdomen for the inflamed animals infused with *anti*-ICAM-1-MBs, while in healthy animals, the mean NRA was 0.43 in the abdominal area; i.e., an approximate doubling (*p* = 0.0188) in the inflamed animals (Fig. 8). The inflammatory stimulus was applied on the left side of the animals, and the MB signals from the right and left sides of the abdomen were quantified separately. The left side showed a mean NRA of 1.00 in the inflamed animals vs. 0.45 in the healthy controls, representing a significant increase in signal intensity on the left side (*p* = 0.0323). The right abdomen gave slightly (not significantly) lower mean MB signals than the left side (inflamed animals NRA 0.69 and healthy animals NRA 0.34), suggesting a higher NRA in inflamed compared with healthy rats (*p* = 0.1418). The differences in MB signals are illustrated in the SPECT/CT hybrid images (Fig. 9), showing a clear signal from the left abdominal area in an inflamed rat and the absence of that signal in a control rat. NRA values illustrating organ distribution are given in Fig. 10. Contrast distribution was also measured as mean relative organ activity (% of injected dose) in control animals *vs.* inflamed animals; lungs 14.4% vs. 5.1%, *p *= 0.0028; liver 31.9% vs. 22.4%, ns; kidneys 4.4% vs. 4.1%, ns; abdomen 3.7% vs. 6.2%, *p *= 0.0272; left abdomen 1.5% vs. 3.6%, *p* = 0.0106; and right abdomen 1.5% vs. 2.4%, ns.

## DISCUSSION

In this study, we showed for the first time that specific antibody coating of our newly developed multimodal MBs caused increased attraction to CAMs on pro-inflammatory cytokine-stimulated human and murine endothelial cells. We also showed sustained attachment of MBs under flow conditions. Furthermore, infusion of *anti*-CAM antibody-labeled MBs in an experimental inflammation model illustrated the feasibility of *in vivo* targeting, and an *ex vivo* study of macrophages in the same model confirmed the specificity of CAM recognition.

### Adhesion Molecules as Strategic Targets

To mimic vascular inflammation, we used a model of pro-inflammatory cytokine stimulation of endothelial cells and confirmed the expression of adhesion molecules by western blotting. Many studies have reported the protein expression patterns for these adhesion molecules, describing basal expression, peak expression, and duration of expression.^17^,^26^,^48^ Peak expression is reportedly reached after 12–24 h cytokine stimulation for CAMs, and after 4–8 h for E-selectin, although some E-selectin expression is seen as early as 2–4 h after cytokine stimulation.^17^,^26^,^48^ We therefore chose 6 h and 24 h activation time points for the different *in vitro* studies. A starvation protocol was applied to the cells to achieve a consistent basal expression level, aiming to synchronize the cells at one stage of their cycle while reducing unwanted effects of serum components. However, serum starvation has been shown to cause cells to undergo apoptosis.^4^ To compensate for this, we added live–dead staining to our flow cytometry analysis, including only live cells in the analysis.

We were able to quantitatively detect a fluorescent signal from our MBs, as measured by flow cytometry and imaged by confocal microscopy, for all three cell types. Our data from flow cytometry (Figs. 2, 3, and 7) together with confocal images (Figs. 4 and 6) showed specific adhesion of greater numbers of targeted MBs adhered to stimulated cells compared with control cells and non-targeted MBs, validating the specificity and targeting properties of the multimodal MB contrast agent. Flow cytometry data showed differences between the degree of adhesion molecule expression and antibody-labeled MBs attachment to MoAoECs and HUVECs, particularly at 24 h stimulation. This may be explained by the methodological procedure of antibody–cell incubation, which was optimized to overcome the physiological effect of floating MBs and to allow MB–cell interactions; this may have led to some degree of activation of untreated cells. Despite this, antibody-labeled MBs (Figs. 2aii and 2bii) seem to follow the same trend overall as antibodies alone (Figs. 2ai and 2bi), and comparisons with non-targeted controls were not affected in the different experimental setups.

To evaluate MB–cell adhesion under flow conditions, we tested MB performance in a low-shear-stress model. Because the duration of antigen–antibody interactions may be limited at higher velocities, we used a laminar flow setup with a maximum flow speed of 9.5 mm s^−1^. For high velocity applications, biotin–streptavidin linked peptides could be used to achieve fast attachment to inflamed endothelium: Klibanov *et al.* have previously shown that peptide-coated contrast agents adhere better than antibody-coated lipid-based MBs under high shear stress.^39^ In their study, firm attachment was defined as a minimum 5-s adherence of MBs to peptide-coated surfaces under flow conditions. Here we showed that adhesion to stimulated cells was maintained even after a high-shear-stress pulse, demonstrating the high avidity of the bond between our antibody-labeled MBs and the adhesion molecules. Attempts at dual targeting have also been made by combining several adhesion molecule targets, possibly increasing the efficiency of adherence under flow.^16^,^25^,^27^,^47^ This is a potential future direction for investigation with our contrast agent.

Monoclonal antibodies coupled to MBs, as in our study, offer high-specificity targeting and allow contrast-enhanced imaging of the targets of interest. In this case approximately 47% of the MB surface area was covered by antibodies, corresponding to ~ 4900 antibodies/*µ*m^2^ or one antibody per 200 nm^2^ of the MB surface, which is at least comparable to antibody conjugated, targeted MBs previously reported (2500 antibodies/*µ*m^2^ or one per 400 nm^2^) by Takalkar *et al.*^67^ The surface area of the MB allows higher numbers of antibodies per MB and that would likely increase the probability of MB–ligand recognition, precision, and accuracy in diagnostic applications.

### Ex Vivo and In Vivo Experiments

It was of interest to evaluate the molecular targeting properties of the *anti*-ICAM-1-labeled MB in both *ex vivo* and *in vivo* conditions. The mouse macrophage or monocyte–macrophage lineage marker F4/80, also known in the rat as epidermal growth factor-like module-containing mucin-like hormone receptor-like 1 (EMR1), can be used as a marker of both monocyte influx and resident peritoneal macrophages.^1^,^78^ Here we showed increased recognition of *anti*-ICAM-1 MBs *ex vivo* by CD11b + EMR1 + macrophages from a rat peritonitis model. Inflammatory cells such as lymphocytes, neutrophils, and monocytes express ICAM-1^19^,^30^,^61^ and are either present or migrate into the peritoneal cavity after Zymosan-induced peritonitis.^21^ In the light of this knowledge and based on our *ex vivo* flow cytometry results (Fig. 7), it is reasonable to hypothesize that the increased signal observed on SPECT imaging of the left abdominal side in the animals with peritonitis is the result of ICAM-1 recognition by *anti*-ICAM-1-labeled MBs. Macrophages may also be present during many different stages of peritonitis, and may therefore be a more suitable target than, e.g., neutrophils, which peak after 24 h and diminish after 48 h.^40^ Ajuebor *et al.* showed an increased influx of greater numbers of blood-derived monocytes compared with polymorphonuclear leukocytes into the peritoneal cavity at 24 h post Zymosan-induced peritonitis.^3^ Yao *et al.* showed that peritoneal mesothelial cells produce cytokines that stimulate the expression of ICAM-1 after activation by Zymosan in rodents.^76^ Anti-inflammatory macrophages and activated mesothelial cells may be present longer than neutrophils,^76^ possibly explaining the observed trend of accumulation of *anti*-ICAM-1-labeled MBs at later time points after Zymosan induction *in vivo*. This speculation could be verified by flow cytometry using additional markers to identify subsets of cells in the peritoneal cavity.

Although MBs as such have the inherent potential for ultrasound imaging, sensitive nuclear techniques are more suited for distribution studies, and a reason to combine the two.^37^ It should also be noted that the comparatively thick shell of these MBs makes them less ideal for traditional ultrasound harmonic imaging techniques, and a special multi-pulse technique for ultrasound imaging of this type of bubbles has been developed (Hansen R, European Patent No. 3125770, Ultrasonic contrast agent detection and imaging). In our *in vivo* experiments comparing animals with peritonitis with healthy control animals, we could quantify an increased signal in inflammatory areas because of accumulation of ^99m^Tc-labeled *anti*-ICAM-1-targeted MBs (Figs. 8 and 9). It has previously been shown that ^99m^Tc-labeled liposomes can detect and image inflamed areas in the abdominal region of rats.^24^,^65^ On the SPECT/CT images, a difference in activity could be noticed on the left side around the cecum of the inflamed animal vs. that in the healthy control (Fig. 9). As anticipated, the MBs were shown to be mainly distributed in the liver and lungs, but some were also present in the kidneys. One explanation for this contrast distribution could be the fact that there are ICAM-1-expressing alveolar macrophages in the lungs and Kupffer cells in the liver.^40^ Our results are consistent with those of previous distribution studies of a similar (but not streptavidin coated) MB in Sprague–Dawley rats, histologically showing intact MBs in lungs, liver and spleen between 10 min and 6 weeks post-injection and in kidneys up to 2 weeks.^5^,^7^ Accumulation of small amounts of free SPIONs was indicated between 2 and 6 weeks in lungs and spleen. Uptake in the kidneys in our study is more counterintuitive because of the size of the MBs, although we cannot exclude the possibility of physical bubble entrapment in the glomeruli, certain bubble degradation or uptake by phagocytic intraglomerular mesangial cells^58^ to explain these findings. Lack of signal in the thyroid indicates that we have very low amounts of free $^{{99{\text{m}}}} {\text{TcO}}_{4} ^{ - }$.

However, uptake by macrophages seems to be a common mode of elimination of PVA MBs of various types, even those not coated with antibodies.^2^,^29^ The fact that macrophages from animals with peritonitis seemed to have a higher affinity for/uptake of *anti*-ICAM-1-MBs compared with control MBs in flow cytometry experiments indicated an effect of the antibodies. Therefore, it is likely that the increased abdominal signal in animals with peritonitis as quantified by SPECT imaging was not only because of abundance of inflammatory macrophages, but also because of increased expression and recognition of ICAM-1 by MBs. The measurement of relative activities in the organs of these animals indicated lower lung and liver signals in the inflamed animals compared with healthy controls, suggesting that the *anti*-ICAM-1-coated MBs tended to gather at sites of inflammation. The multimodal contrast agent studied in this paper serves as an experimental prototype for further understanding and investigation of multifunctional contrast agents for diagnostic applications.

Biocompatible materials such as PVA and SPIONS are widely used and applied in various types of contrast agents.^52^,^54^ The long-term retention of these non-biodegradable materials *in vivo* may cause unwanted side effects.^35^,^44^,^46^,^66^ However, two SPION-based contrast agents are available for clinical use i.v.; ferucarbotran (Resovist^®^), coated with carboxydextran and used for contrast enhanced liver imaging^60^,^70^; and ferumoxytol coated with a semi-synthetic carbohydrate and approved by the Food and Drug Administration (FDA) for treatment of iron deficiency in patient with renal failure, and off-label use as an MRI-contrast agent.^50^,^71^ The limitations that come with potential long-term toxicity must also be weighed against the clinical benefits of diagnostic imaging and treatment. At present there is ongoing effort to develop more safe and tolerated SPION-based contrast agents.^35^,^44^,^46^,^54^ Thus, optimal concentration and coating material need to be taken into consideration in contrast development utilizing SPIONs and non-biodegradable materials to minimize long-term adverse effects.

A challenge for future contrast development is to prolong the circulation time of the agents, including limiting their elimination by the immune and complement systems and minimizing rapid clearance from the circulation, thereby improving their chances of reaching target sites. Additionally the buildup of a “protein corona,” mainly consisting of serum albumin, as was shown for similar MBs by Wan *et al.*,^62^,^69^ could potentially improve the ability of the MBs to reach the target organ or cells of interest by prolonging their half-life in blood circulation, although their binding sites may be less accessible. A streptavidin linkage, as used in our MBs, has been used in many theranostic approaches because of its high tolerance to heat and denaturation, in addition to its strong binding affinity to biotin. However, it is well known that this protein, which is derived from *Streptomyces avidinii*, can trigger an immunogenic response to the charged and aromatic residues on its protein surface, which limits its clinical applications. Yumura *et al.* reported a method for reducing the immunogenicity of nonhuman proteins, which may present a solution to this problem and potentially broaden the application of this contrast agent beyond preclinical use.^77^

We here present a versatile multimodal, targeted MB with potential for specific diagnostic applicability in the imaging field. An advantage of MBs as contrast agents is their high surface coverage of the targeting motif, which increases their targeting efficacy and the likelihood that the contrast agent will interact with its target. Our findings open up the future possibility of drug delivery targeted towards inflammatory diseases using an antibody-coated drug carrier, e.g., a modification of our MBs.^51^ Some studies even suggest that blocking antibodies towards e.g., CAMs, may have therapeutic applications,^48^ widening the applicability of these types of contrast agents.

## CONCLUSION

We have demonstrated that our new contrast agent has the capacity for specific targeting *in vitro*, as well as the potential for *in vivo* imaging for research purposes in animal models. Our multimodal targeting MB permits high ligand surface density and allows selective targeting of inflammatory markers such as ICAM-1, VCAM-1, and E-selectin, providing a new tool for multiple modes of diagnostic molecular imaging of inflammation. Combining targeting with the versatile applications of this contrast agent as a tool for experimental studies generated proof of principle. The MB construction and its thick LbL-PVA shell of several hundred nanometers also allow further exploitation for therapeutic purposes by incorporation of drugs. However, more studies *in vivo* are needed to further evaluate these characteristics.

## Electronic supplementary material

Below is the link to the electronic supplementary material.

Supplementary material 1 (TIFF 565 KB). A) ELISA showing OD-values of excessive antibodies from MB-Ab-conjugation washes. B) Micro plate BCA protein assay showing measured amount of antibodies (0.1, 0.2, 0.7, 8.5 and 11.8 μg) per 10^8^ MBs from the same solution as in A), when 0, 4, 6, 12 and 20 *μ*g of antibodies were added per 10^8^ MBs. Saturation of bound antibodies was reached between 6 and 12 μg of added antibodies per 10^8^ MBs. Values are presented as mean ± SD (n = 4 for all data points)

Supplementary material 2 (TIFF 5849 KB). Western blot showing increased protein expression of ICAM-1, VCAM-1, and E-selectin after cytokine stimulation of A) MoAoECs with both TNF-α and IL-1β for 6 h and 24 h, and B) HUVECs with TNF-α for 6 h and 24 h. Loading controls: human β-actin for HUVECs and mouse α-tubulin for MoAoECs

Supplementary material 3 (TIFF 4761 KB). Flow cytometry plots showing examples of gating strategies for A) MoAoECs and B) HUVECs incubated with either antibodies or MBs. Graphs show antibody targeting of adhesion molecules on both untreated and cytokine-treated cells. Results are presented as histograms of fluorescence intensities (FI)

Supplementary material 4 (Mov 49658 KB). SUPPLEMENTARY VIDEO. Video of anti-ESEL MB adhesion to stimulated HUVECs under flow at 2 dyn cm^−2^
