# Supplementary figures and images for: Molecular Imaging of a New Multimodal Microbubble for Adhesion Molecule Targeting
Source: Cell Mol Bioeng. 2018 Nov 28;12(1):15–32. doi: 10.1007/s12195-018-00562-z (PMC6816780; doi:10.1007/s12195-018-00562-z)

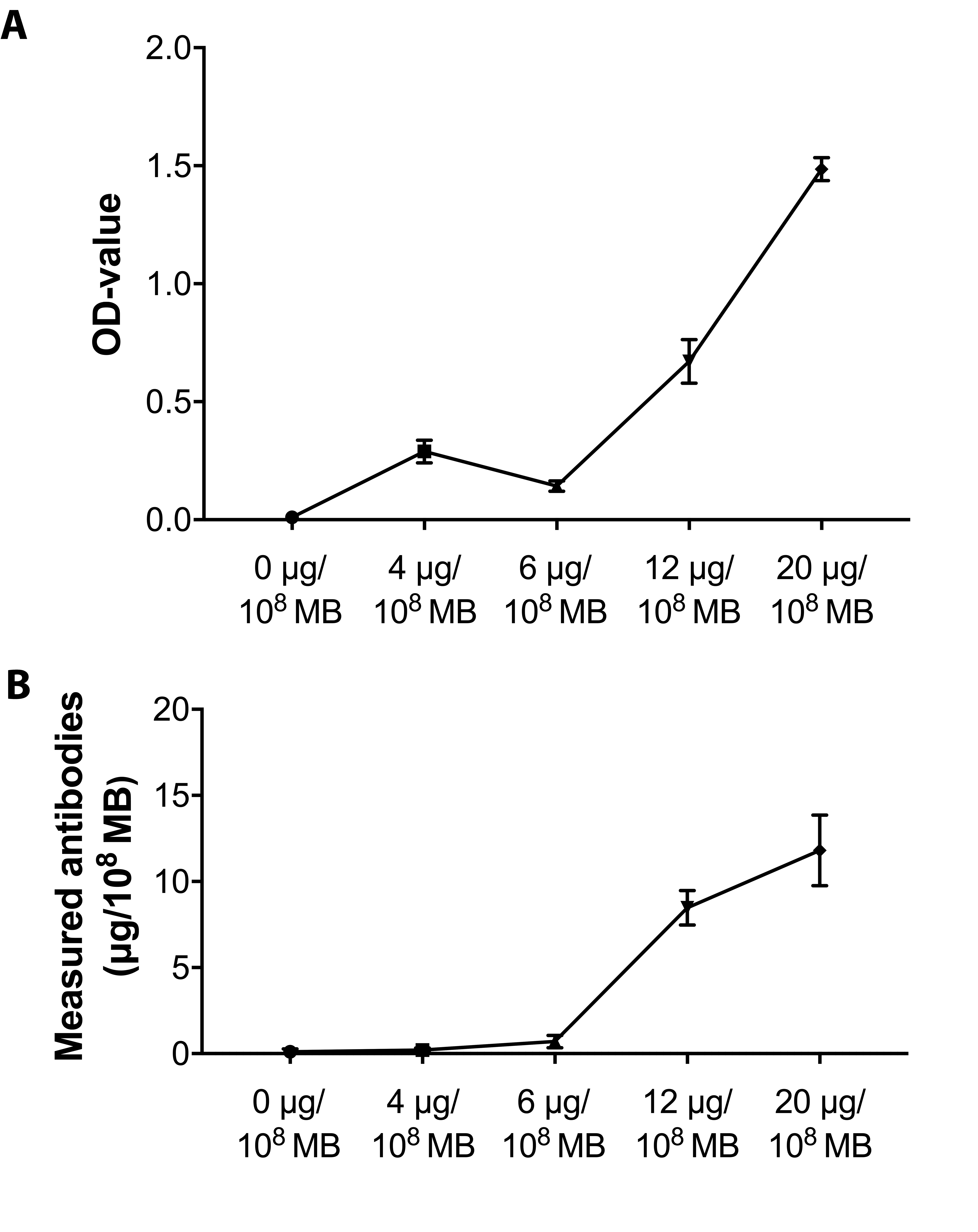

Supplement: Supplementary file 1 — Supplementary material 1 (TIFF 565 KB). A) ELISA showing OD-values of excessive antibodies from MB-Ab-conjugation washes. B) Micro plate BCA protein assay showing measured amount of antibodies (0.1, 0.2, 0.7, 8.5 and 11.8 μg) per 108 MBs from the same solution as in A), when 0, 4, 6, 12 and 20 μg of antibodies were added per 108 MBs. Saturation of bound antibodies was reached between 6 and 12 μg of added antibodies per 108 MBs. Values are presented as mean ± SD (n = 4 for all data points) [file 12195_2018_562_MOESM1_ESM.tif]

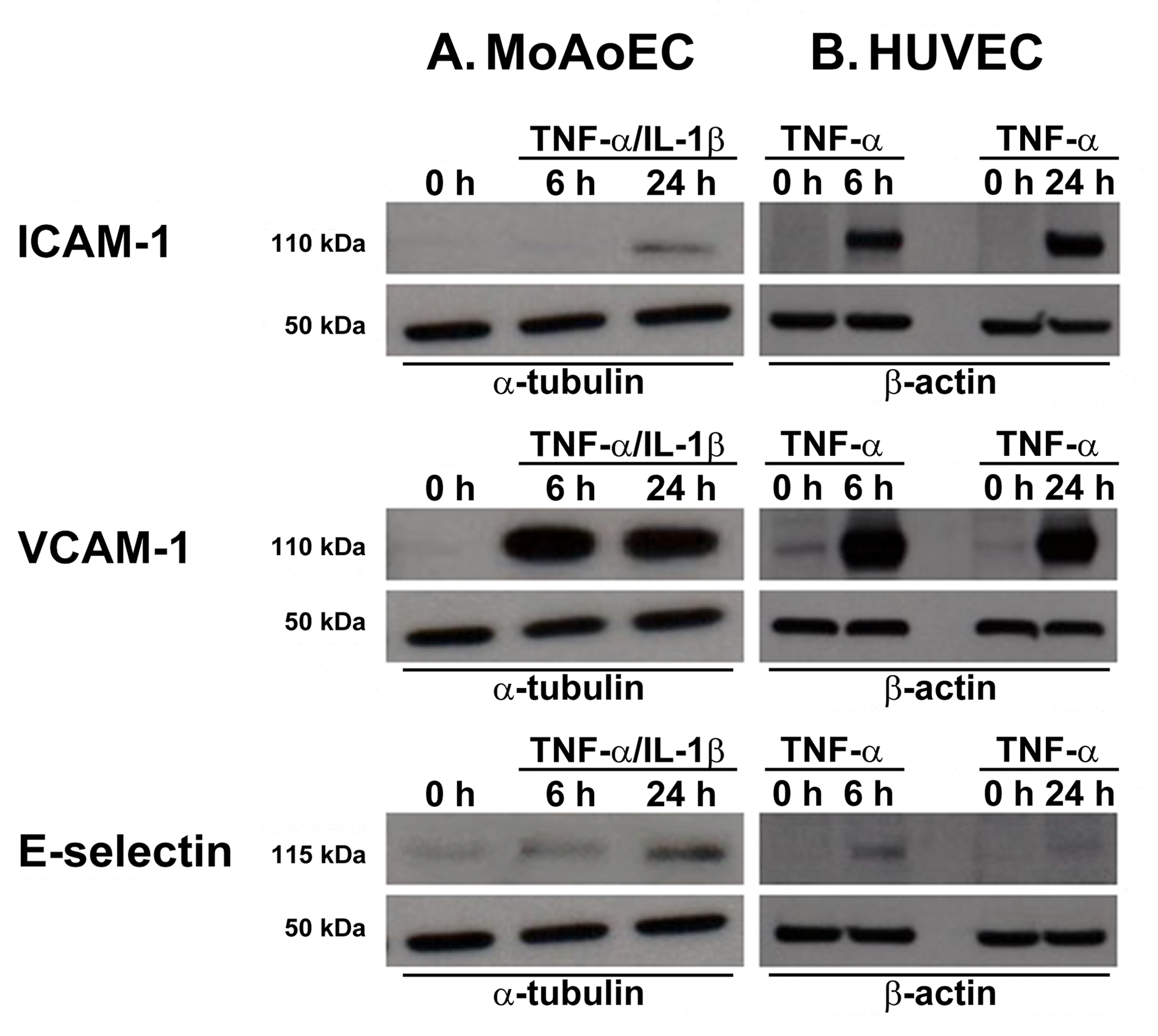

Supplement: Supplementary file 2 — Supplementary material 2 (TIFF 5849 KB). Western blot showing increased protein expression of ICAM-1, VCAM-1, and E-selectin after cytokine stimulation of A) MoAoECs with both TNF-α and IL-1β for 6 h and 24 h, and B) HUVECs with TNF-α for 6 h and 24 h. Loading controls: human β-actin for HUVECs and mouse α-tubulin for MoAoECs [file 12195_2018_562_MOESM2_ESM.tif]

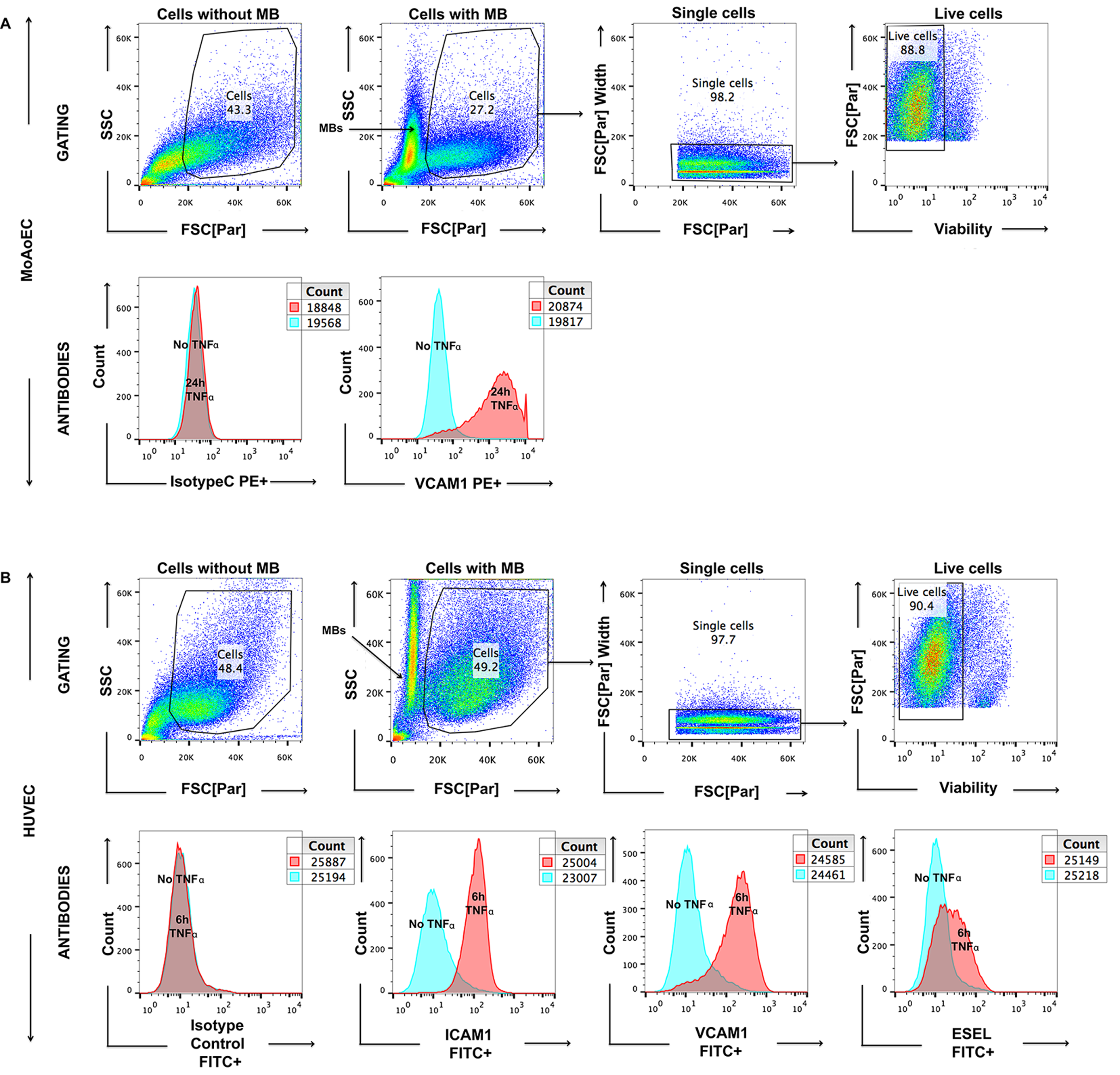

Supplement: Supplementary file 3 — Supplementary material 3 (TIFF 4761 KB). Flow cytometry plots showing examples of gating strategies for A) MoAoECs and B) HUVECs incubated with either antibodies or MBs. Graphs show antibody targeting of adhesion molecules on both untreated and cytokine-treated cells. Results are presented as histograms of fluorescence intensities (FI) [file 12195_2018_562_MOESM3_ESM.tif]
